# Evaluation of biometric indicators of anterior segment parameters after ICL implantation by swept-source optical coherence tomography

**DOI:** 10.1186/s12886-023-02942-0

**Published:** 2023-05-02

**Authors:** Chuhao Tang, Tong Sun, Zhengze Sun, Hongyu Duan, Yilin Liu, Lu Zhao, Wenlong Li, Linbo Bian, Hong Qi

**Affiliations:** Department of Ophthalmology, Peking University Third Hospital, Beijing Key 9 Laboratory of Restoration of Damaged Ocular Nerve, Beijing, China

**Keywords:** Implantable collamer lens implantation surgery, Myopia, Optical coherence tomography, Vault, Iridotrabecular contact

## Abstract

**Background:**

To evaluate anterior segment structural alterations after implantable collamer lens (ICL) implantation in myopic patients using swept-source quantitative optical coherence tomography (SS-OCT).

**Methods:**

This prospective study included 47 eyes in 24 patients with preoperative spherical equivalent ≥ -3.00 D. Patients underwent ICL implantation at Department of Ophthalmology, Peking University Third Hospital, from May 2021 to December 2022. SS-OCT was used to measure anterior chamber width (ACW), angle opening distance (AOD), angle recess area (ARA), trabecular-iris area (TISA), trabecular-iris angle (TIA), iridotrabecular contact (ITC) area, and ITC Index before ICL implantation surgery and at 1 month follow-up. The correlations among the ITC index, vault, and angle parameters were analysed. Receiver operating characteristic (ROC) analysis was used to explore the ability of the vault to identify eyes with suspected angle-closure.

**Results:**

At one month following ICL implantation, the ITC area was 0.396 ± 0.37 mm^2^, and the ITC index is 8.143 ± 5.439%. All angle parameters, except ACW, showed a statistically significant reduction on SS-OCT (*P* < 0.05). Mean AOD500, AOD750, ARA500, ARA750, TISA500, TISA750, TIA500, and TIA750 values at one month postoperatively decreased by 60.0%, 60.4%, 58.1%, 59.2%, 57.3%, 58.7%, 48.8%, and 50.7%, respectively. The vault was positively correlated with the ITC index and percent change in anterior chamber angle parameters. A vault of > 0.659 mm was found to be optimal for angle-closure suspect with a sensitivity of 85.2% and a specificity of 53.9%.

**Conclusions:**

Anterior chamber angle parameters decreased one month after ICL implantation, and their percentage changes and ITC index correlated with the vault. When the vault is larger than 0.659 mm, it is necessary to be alert to possible closed angle suspicion.

## Introduction

Uncorrected refractive error is the third leading cause of blindness and the first cause of moderate to severe visual impairment worldwide [1]. Myopia is the most common type of refractive error, and uncorrected myopia costs hundreds of billions of dollars in productivity loss worldwide each year [2]. As a kind of intraocular refractive surgery, implantable collamer lens (ICL; Staar Surgical, Nidau, Switzerland) implantation has been widely affirmed for its safety and effectiveness in correcting moderate and high myopia [3, 4]. However, the intraocular lens is implanted into the posterior ciliary sulcus during ICL implantation, which has a particular impact on the anatomical structure of the anterior segment and may affect the drainage of aqueous humor due to narrowing or closure of the anterior chamber angle (ACA), resulting in elevated eye pressure or secondary glaucoma [5–7].

Anterior segment optical coherence tomography (AS-OCT) is a new, non-invasive, high resolution, cross-sectional imaging technique with broad application potential in the anterior segment. Swept-source OCT (SS-OCT; CASIA2; Tomey, Nagoya, Japan) is a type of AS-OCT which performs 128 meridional scans and analysis with a high-speed A-scan in 2.4 s for a 360° ACA [8]. Its built-in software can be used to determine the 360° iridotrabecular contact area (ITC area) and calculate the ITC index. After ICL implantation, the anterior chamber depth (ACD) becomes shallower, and the ACA becomes narrower, increasing the risk of peripheral anterior synechia. SS-OCT parameters, including the ITC index, may characterise the extent of angle-closure and other biometric parameters that reflect the mechanical obstruction of the aqueous outflow pathway. Therefore, this study aimed to investigate the correlation between the ITC index and anterior chamber parameters, angle parameters, vault, and intraocular pressure after ICL implantation, and whether it can be used as an indicator to evaluate the risk of angle-closure glaucoma. To the best of our knowledge, this is the first study to explore the risk of angle-closure or angle-closure glaucoma after ICL implantation in combination with the ITC index, ACA parameters, and vault.

## Methods

### Participants

This study included 47 eyes in 24 participants with moderate and high myopia. All patients underwent ICL implantation surgery at the Department of Ophthalmology, Peking University Third Hospital between May 2021 and December 2022. Inclusion criteria: 21–45 years old, with a spherical equivalent (SE) of greater than − 3.00 diopters (D), ACD ≥ 2.8 mm, corneal endothelial cell count (cECC) ≥ 2000 cells/mm^2^, SE remained unchanged for more than 1 year. None of the patients included in this study had a history of intraocular surgery and had any other ocular pathologies (e.g., uveitis, glaucoma, cataract, keratoconus, or severe dry eye) or serious systemic diseases (e.g., diabetes, uncontrolled hypertension, or severe hyperthyroidism).

Before surgery, each participant underwent a full ocular examination, and the horizontal white-to-white (WTW) distance and axial length (AL) were measured by optical biometry (IOL Master 700; Carl Zeiss Meditec, Jena, Germany). cECC was obtained from each eye using a corneal endothelial microscope (SP-2000; Topcon, Tokyo, Japan). Additionally, each eye was subjected to slit-lamp biomicroscopyic examination, corneal topography and funduscopic examination.

Moreover, each patient’s eye was assessed for uncorrected visual acuity (UCVA), best-corrected visual acuity (BCVA), intraocular pressure (IOP), and manifest refraction before and one month afterward. For statistical analysis, the decimal Snellen evaluation of UCVA and BCVA was converted to the logarithm of the minimum angle of resolution (logMAR). The aid using a non-contact tonometer (CT-80; Topcon, Tokyo, Japan), the IOP was measured. The central vault of the ICL (distance from the posterior surface of the ICL to the crystalline lens) was measured using AS-OCT one month after surgery.

The size of the ICL was calculated using the STAAR sizing formula based on the results of WTW and ACD. The myopic patients were scheduled for standard ICL implantation surgeries by the same surgeon (HQ) under similar settings. Preoperatively, in all patients, topical anaesthesia (4% lidocaine) was administered 30 min preoperatively. Through a 3.0-mm temporal corneal incision, the ICL was slowly inserted into the anterior chamber following the implantation of hyaluronic acid (ViscAid, Beijing, China) under visualisation with an OPMI Lumera 700 surgical microscope (Carl Zeiss Meditec, Germany). The Toric ICL implantation surgery was completed using the Callisto Eye System (Carl Zeiss Meditec, Germany). Any remaining viscosurgical devices were washed out of the anterior chamber with a balanced salt solution. Antibiotics, steroids, and artificial tear drops were administered postoperatively.

### Swept-source optical coherence tomography

All participants underwent CASIA2 SS-OCT imaging indoors under natural light before any contact procedure. The participants assumed a sitting position, placed their chin on the chin rest, and adjusted the position of the eyes until they could see the scanning direction of the aiming light. The operator adjusts the area of the eye visible in the iris viewport until the iris is in sharp focus. The controls are then used to make coarse adjustments and move the jaw rest until the corneoscleral junction was in view. Finally, the scleral spur (SS) was noted in each scan by a single observer with relevant experience and training who was blinded to the gonioscopic findings.

**Fig. 1:**
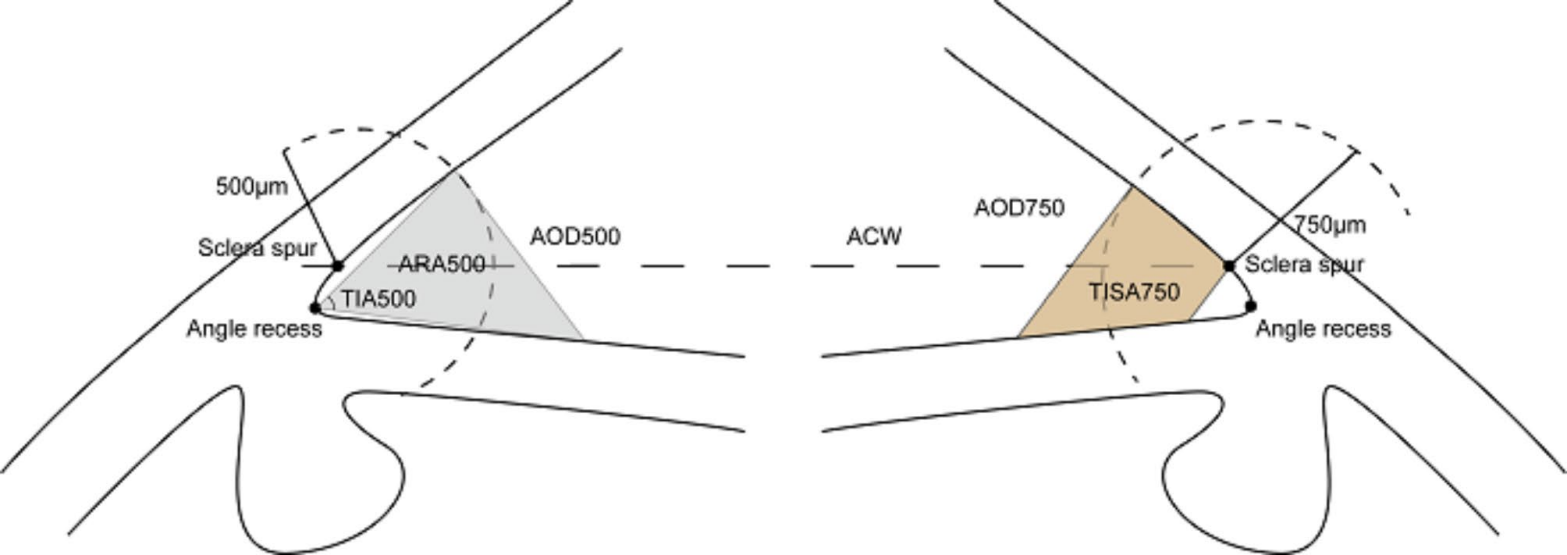
Anterior chamber angle parameters measured with SS-OCT. ACW, anterior chamber width; AOD, angle opening distance; ARA, angle recess area; TISA, trabecular iris area; TIA, trabecular-iris angle

**Fig. 2:**
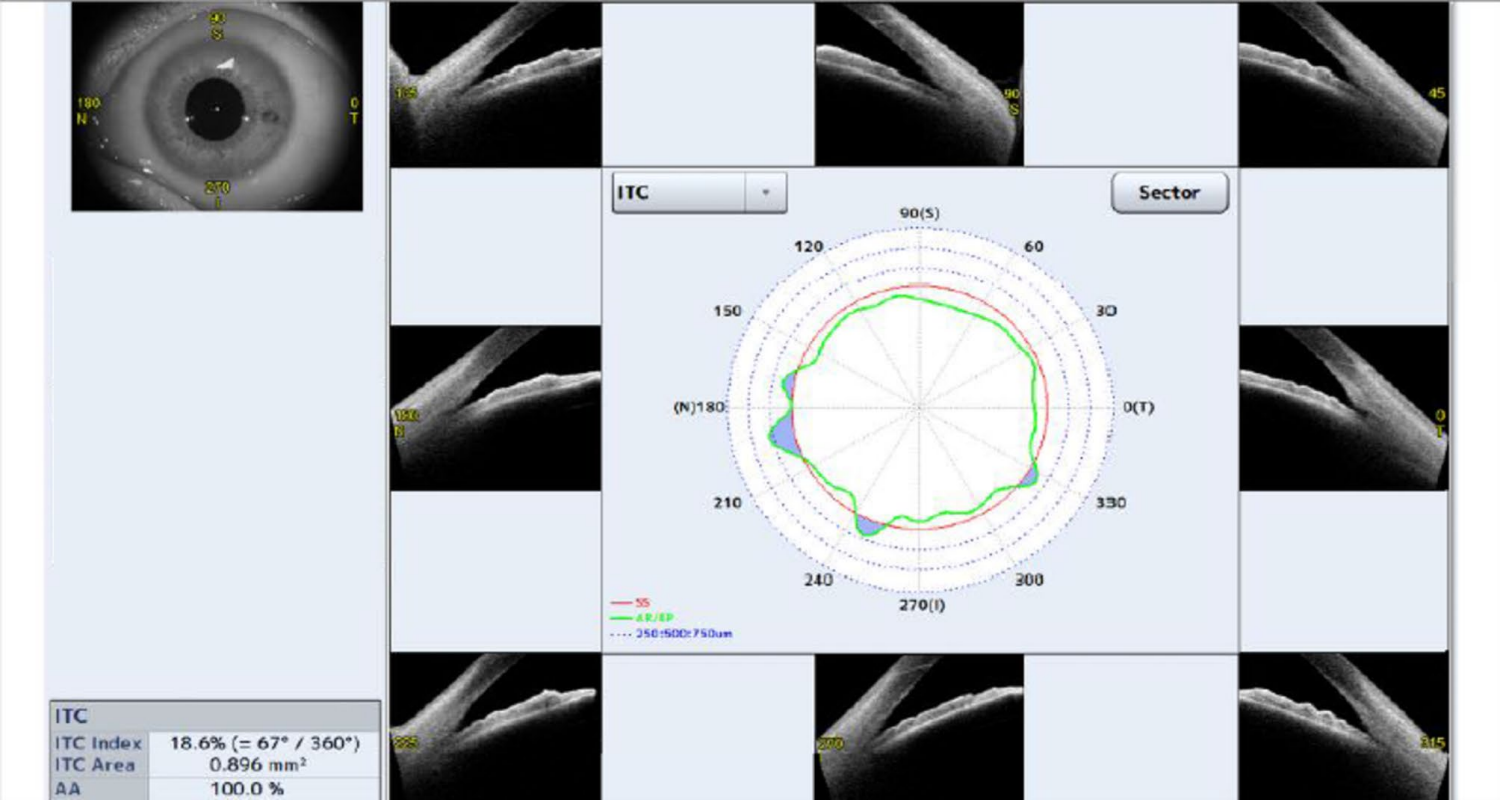
SS-OCT image showing the ITC parameter analysis

The quantitative parameters of SS-OCT were as follows (Fig. 1):

1. ACD: The distance between the posterior surface of the cornea and the anterior surface of the crystalline lens.
2. Anterior Chamber Width (ACW): The distance between two SS in the OCT scan image.
3. Angle Opening Distance (AOD): The trabecular mesh at 500 μm away from the SS is drawn as a vertical line perpendicular to the posterior surface of the cornea and intersects with the iris. The distance between the two points was AOD500; the trabecular mesh at 750 μm away from the SS draws a vertical line perpendicular to the posterior surface of the cornea and intersects with the iris. The distance between the two points was AOD750.
4. Angle Recess Area (ARA): ARA500 is the triangular area bounded by AOD500 (base) and the angle recess (apex); the triangular area bounded by AOD750 (base) and the angle recess (apex) is ARA750.
5. Trabecular-Iris Area (TISA): TISA500 refers to the area enclosed by the anterior surface of the iris, AOD500, a line drawn from the SS perpendicular to the plane of the inner scleral wall to the opposing iris and the posterior surface of the cornea; and TISA750 refers to the area enclosed by the anterior surface of the iris, AOD750, a line drawn from the SS perpendicular to the plane of the inner scleral wall to the opposing iris and the posterior surface of the cornea.
6. Trabecular-Iris Angle (TIA): The angle between the two endpoints of AOD500 and the line connecting the angle recess is TIA500, and the angle between the two ends of AOD750 and the line connecting the angle recess is TIA750.
7. Iridotrabecular Contact Area (ITC Area): Contact area between the iris and the wall angle anterior to the SS.
8. Iridotrabecular Contact Index (ITC index): The inbuilt semiautomated software of the CASIA can be used to measure the ITC index, which is a quantitative measure of the extent of angle-closure across 360° of the angles expressed as a percentage (Fig. 2). ITC analysis uses anterior segment cross-sectional meridional images to determine the extent of contact between the iris and angle wall [8].

### Statistical analysis

SPSS Statistics version 26.0 (IBM Corp., Chicago, IL, USA) and R version 4.2.2 (The R Foundation for Statistical Computing) were used to conduct all statistical analyses. Normality of the data distributions was assessed using the Shapiro–Wilk test. All descriptive statistics were summarised and displayed as the mean ± standard deviation or the median (25–75%). Continuous variables and normally distributed data were compared using independent sample t-tests. In addition, continuous variables that were not normally distributed were compared using the Mann–Whitney U test. The correlations between different parameters were assessed using Pearson’s or Spearman’s rank correlations. Receiver operating characteristic (ROC) analysis was used to explore the ability of the vault to identify eyes with suspected angle-closure. Statistical significance was set at *P* < 0.05.

## Results

The demographic characteristics of the enrolled participants are summarised in Table 1. The mean patient age at the time of surgery was 28.33 ± 5.58 years (range, 21–42 years). The preoperative manifest refraction spherical equivalent (MRSE) was − 7.66 ± 3.11 (range, -3.50 to -15.00 D). The preoperative manifest sphere was − 7.24 D ± 3.03 D (range, − 3.00 to − 14.25 D). The preoperative manifest refractive cylinder was − 0.84 ± 0.52 D (range, 0.00 to − 2.50 D). The AL was 26.32 ± 1.55 mm (range, 24.40–28.82 mm), WTW was 11.85 ± 0.24 mm (range, 11.3–12.3 mm), and ACD was 3.23 ± 0.22 mm (range, 2.89–3.76 mm).

**Table 1:** Preoperative demographics of myopic patients who underwent implantable collamer lens implantation in this study

Characteristic	Mean ± SD
Number, people/eyes	24/47
Sex, male/female	4/20
Age (years)	28.33 ± 5.58 (range 21 to 42)
MRSE (D)	−7.66 ± 3.11(range − 3.50 to -15.00)
LogMAR BCVA	0.006 ± 0.019
AL (mm)	26.32 ± 1.55
WTW (mm)	11.85 ± 0.24
ACD (mm)	3.23 ± 0.22
cECC ($\text{c}\text{e}\text{l}\text{l}\text{s}/{\text{mm}}^{\text{2}}$)	2903.73 ± 272.03
ICL size (mm)	12.6 ± 0.28
ICL power (D)	-8.19 ± 3.45 (range − 4.00 to -15.50)

MRSE, manifest refraction spherical equivalent; LogMAR, logarithm of the minimum angle of resolution; BCVA, best-corrected visual acuity; AL, axial length; WTW, white-to-white; ACD, anterior chamber depth; cECC, corneal endothelial cell count

**Fig. 3:**
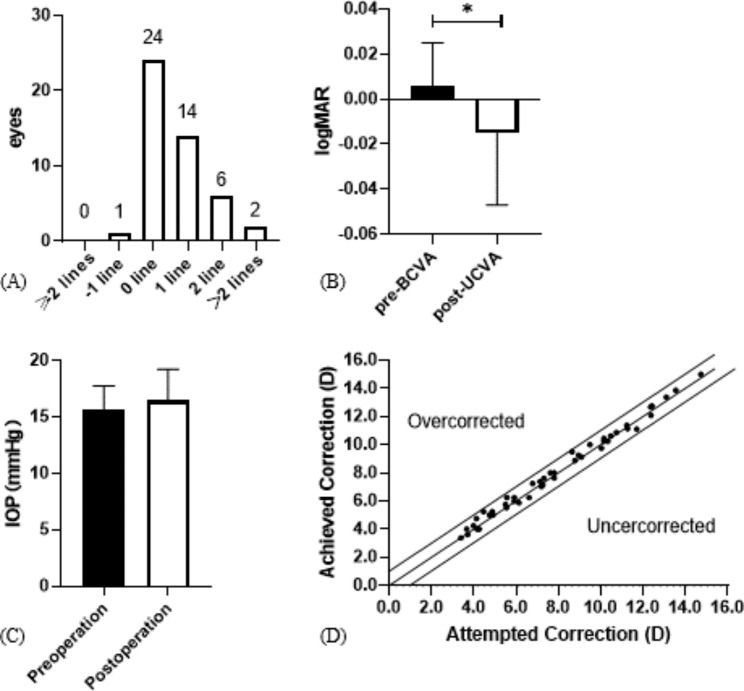
Clinical examinations of myopic patients after ICL implantation surgery. (**A**) Changes in Snellen lines of BCVA at 1 month after ICL implantation. (**B**) Changes between UCVA 1 month after ICL implantation and BCVA. (**C**) Changes in intraocular pressure 1 month after ICL implantation. (**D**) A scatter plot of the attempted versus the achieved manifest spherical equivalent correction 3 months after ICL implantation. * *P* < 0.05 versus preoperative

All surgical procedures were uneventful, and no postoperative complications, such as cataract formation, pigment dispersion syndrome, pupillary block, or axis rotation, were observed throughout the observation period. Visual acuity improved in all the patients on the first postoperative day. One month postoperatively, only one eye (2.13%) lost one line of visionm and 46 eyes (97.87%) maintained or improved BCVA (Fig. 3A). The efficacy index was 1.06 ± 0.0.09 (preoperative BCVA: 0.006 ± 0.019 logMAR and postoperative UCVA: -0.0015 ± 0.0032 logMAR, *P* < 0.05; Fig. 3B). The mean preoperative IOP and postoperative IOP (15.58 ± 2.15 versus 16.44 ± 2.77, *P* > 0.05; Fig. 3C) were not significantly different. At one month postoperatively, 91% and 100% were within ± 0.50 and 1.00 D of the attempted correction, respectively (Fig. 3D).

The iridotrabecular Contact and Anterior Segment SS-OCT Parameters were assessed at baseline and one month postoperatively (Table 2). At one month following ICL implantation, the vault was 0.519 ± 0.244 mm, the ITC area was 0.396 ± 0.37 mm^2^, and the ITC index was 8.143 ± 5.439%. All angle parameters, except ACW, showed a statistically significant reduction on SS-OCT (*P* < 0.05). Mean AOD500, AOD750, ARA500, ARA750, TISA500, TISA750, TIA500, and TIA750 values at one month postoperatively decreased by 60.0%, 60.4%, 58.1%, 59.2%, 57.3%, 58.7%, 48.8%, and 50.7%, respectively.

**Table 2:** Iridotrabecular Contact and Anterior Segment SS-OCT Parameters of ICL patients pre- and postoperatively (Mean ± SD)

	Preoperatively	Postoperatively
ACW (mm)		
Horizontal	11.663 ± 0.282	11.747 ± 0.298
Vertical	12.004 ± 0.369	11.927 ± 0.361
Average	11.889 ± 0.289	11.837 ± 0.305
AOD500 (mm)		
Nasal	0.754 ± 0.286	0.271 ± 0.095*
Temporal	0.853 ± 0.398	0.294 ± 0.102*
Superior	0.675 ± 0.343	0.25 ± 0.091*
Inferior	0.798 ± 0.372	0.297 ± 0.093*
Average	0.77 ± 0.316	0.278 ± 0.077*
AOD750 (mm)		
Nasal	0.979 ± 0.291	0.346 ± 0.128*
Temporal	1.087 ± 0.409	0.385 ± 0.133*
Superior	0.905 ± 0.377	0.333 ± 0.113*
Inferior	1.013 ± 0.378	0.396 ± 0.122*
Average	0.996 ± 0.332	0.365 ± 0.105*
ARA500 (mm^2^)		
Nasal	0.273 ± 0.13	0.105 ± 0.041*
Temporal	0.346 ± 0.253	0.116 ± 0.048*
Superior	0.237 ± 0.144	0.093 ± 0.044*
Inferior	0.325 ± 0.201	0.114 ± 0.04*
Average	0.295 ± 0.152	0.107 ± 0.031*
ARA750 (mm^2^)		
Nasal	0.487 ± 0.195	0.182 ± 0.068*
Temporal	0.581 ± 0.35	0.199 ± 0.076*
Superior	0.434 ± 0.226	0.163 ± 0.072*
Inferior	0.546 ± 0.292	0.201 ± 0.063*
Average	0.512 ± 0.231	0.187 ± 0.055*
TISA500 (mm^2^)		
Nasal	0.255 ± 0.115	0.1 ± 0.037*
Temporal	0.306 ± 0.187	0.111 ± 0.042*
Superior	0.226 ± 0.135	0.089 ± 0.039*
Inferior	0.292 ± 0.165	0.107 ± 0.036*
Average	0.27 ± 0.129	0.102 ± 0.028*
TISA750 (mm^2^)		
Nasal	0.47 ± 0.183	0.177 ± 0.065*
Temporal	0.542 ± 0.286	0.194 ± 0.07*
Superior	0.423 ± 0.218	0.159 ± 0.067*
Inferior	0.513 ± 0.258	0.195 ± 0.059*
Average	0.487 ± 0.208	0.181 ± 0.052*
TIA500 (deg.)		
Nasal	60.594 ± 15.948	29.578 ± 12.842*
Temporal	59.531 ± 16.145	30.356 ± 10.016*
Superior	58.006 ± 24.231	26.778 ± 7.351*
Inferior	58.541 ± 20.916	28.453 ± 7.758*
Average	59.168 ± 16.755	28.791 ± 6.944*
TIA750 (deg.)		
Nasal	55.352 ± 12.355	24.948 ± 8.901*
Temporal	55.018 ± 12.908	27.179 ± 8.8*
Superior	53.733 ± 20.866	24.573 ± 6.277*
Inferior	54.309 ± 16.465	26.212 ± 6.909*
Average	54.603 ± 13.886	25.728 ± 6.237*
ITC area (mm^2^)		0.396 ± 0.37
ITC index (%)		8.143 ± 5.439
Vault (mm)		0.519 ± 0.244

ACW, anterior chamber width; AOD, angle opening distance; ARA, angle recess area; TISA, trabecular iris area; TIA, trabecular-iris angle; ITC, iridotrabecular contact. * *P* < 0.05 versus preoperative values

**Fig. 4:**
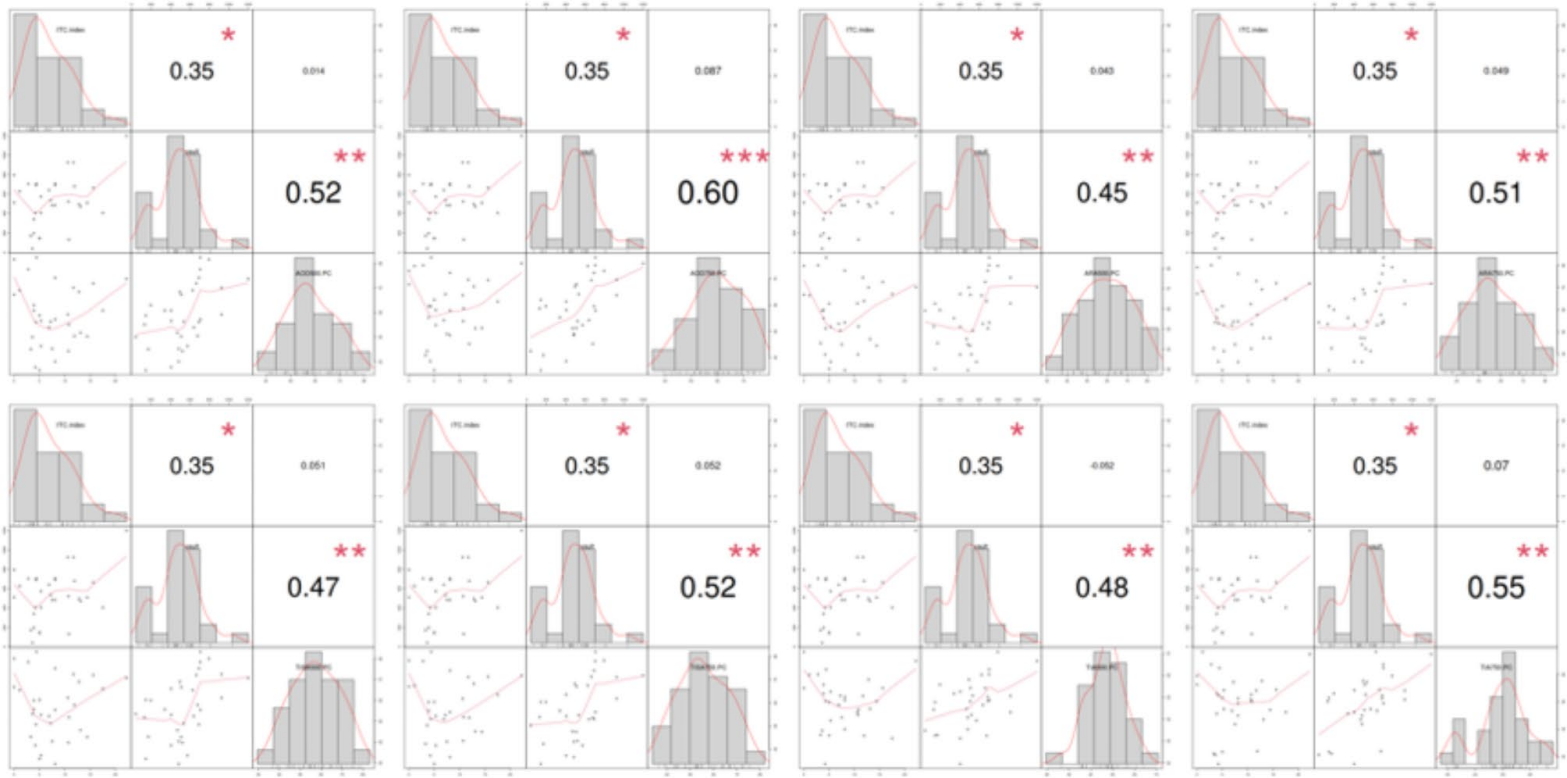
Correlation analysis of percentage changes of anterior chamber angle parameters and ITC index and vault after ICL implantation surgery. AOD, angle opening distance; ARA, angle recess area; TISA, trabecular iris area; TIA, trabecular-iris angle. PC, percentage changes; * *P* < 0.05; ** *P* < 0.01

A correlation analysis examining the relationships between the ITC parameters and other factors was conducted. The ITC index was positively correlated with the vault one month after ICL implantation, but no relationship was found between the ITC index and IOP, angle parameters, or percentage change in angle parameters. However, the vault value after ICL implantation was significantly and positively correlated with the percentage change in angle parameters, such as AOD500, AOD750, ARA500, ARA750, TISA500, TISA750, TIA500, and TIA750(Fig. 4).

**Fig. 5:**
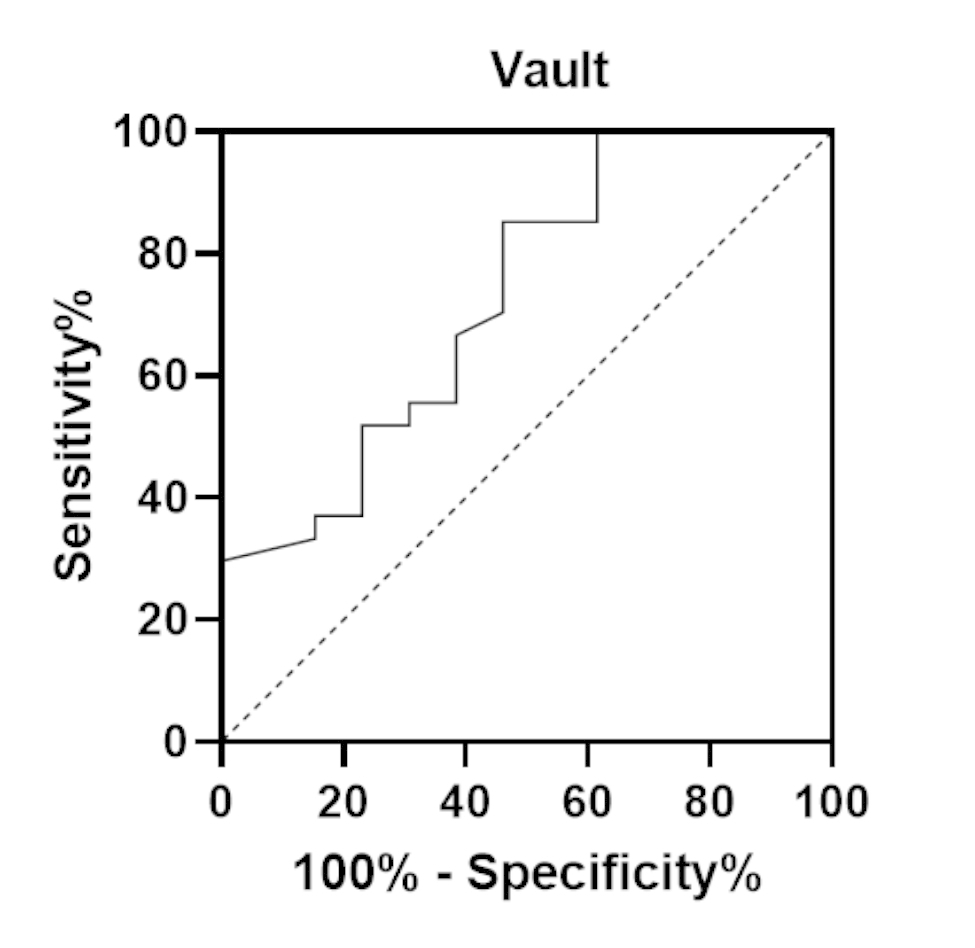
Receiver operating characteristic curve analyses for the vault identifying eyes with angle-closure suspect

Receiver operating characteristic curves, with area under the curve (AUC) and 95% confidence intervals (CIs), were used to assess the performance of the vault for identifying eyes with angle-closure suspicion, taking the ITC index ≥ 10.76% as the reference standard (Fig. 5). The AUC was 0.73 (95% CI, 0.56–0.90; *P*<0.05). The vault of > 0.659 mm was found to be optimal for angle-closure suspect with a sensitivity of 85.2% (95% CI, 67.5–94.1) and a specificity of 53.9% (95% CI, 29.1–76.8).

## Discussion

ICL implantation is an important method for correcting myopia. Compared with corneal refractive surgery, ICL implantation has good safety, stability, and reversibility [9, 10]. It does not cut the cornea, while retaining lens accommodation. The latest generation of ICL V4c drains the aqueous humor through the central hole, thus solving the shortcomings of traditional ICL surgery, which requires peripheral iridectomy and reduces damage to the ocular structure. Many studies have shown that ICL implantation can effectively improve postoperative visual acuity and quality [11, 12]. Based on our data, ICL implantation is a safe and effective treatment for myopia. There were no postoperative complications throughout the observation period, and the efficacy index was 1.06 ± 0.0.09. None of the eyes lost two or more lines of vision after ICL implantation. All eyes were within ± 1.00 D of the attempted correction and 91% of eyes were within ± 0.50 D of the attempted correction. However, when an ICL is implanted into the eye, the loop foot is fixed to the ciliary sulcus of the posterior chamber, which inevitably contacts the posterior surface of the iris. Therefore, examination of the chamber angle structure is important to ensure safety after ICL implantation.

In this study, SS-OCT was primarily used to examine angle parameters following ICL implantation. At present, the commonly used equipment for observing the chamber angle includes gonioscopy, ultrasound biomicroscopy (UBM), the Pentacam anterior segment analysis system, and OCT [13]. Gonioscopy and UBM are contact examinations that are not suitable for patients in the early postoperative period, and the measurement results depend on the technique and subjective judgement of clinicians. The Pentacam uses a Scheimflug camera to scan and shoot, which cannot directly visualise the angular structure because visible light cannot penetrate deep into the tissue. The SS-OCT used in this study is a non-contact rapid anterior segment imaging device with fast imaging speed, high resolution, and good signal-to-noise ratio. It can automatically analyse the image and obtain the position of the scleral protrusion and angle recess; obtain parameters, such as AOD, ARA, TISA, TIA, and the proprietary ITC index; and conduct a fully automatic analysis and statistics of the opening and closing of the chamber angle.

The ACA is an important basis for evaluating risk factors for glaucoma and changes in the anterior segment. In 2001, Abela-Formanek et al. [14] first used gonioscopy and found that approximately 58% of eyes with ACA less than 20 ° after ICL implantation. Fernández-Vigo et al. [15] reported in 2017 that ACA decreased by 39–45% at 1 month after ICL V4c implantation using FD-OCT and remained stable for the next 2 years. Liu et al. [16] used SS-OCT to observe that AOD500, TISA500, and TIA500 decreased by 65.4–71%, 64.1–69.3%, and 53.8–61.5% after ICL V4c implantation. Therefore, we evaluated ACA alterations one month after ICL implantation in myopic patients using SS-OCT. The reductions in the ACA parameters observed in our study were similar to those reported in previous studies. We also found that the vault was positively correlated with the percent change in anterior chamber parameters, including the ACA, but not with anterior chamber parameters, after ICL implantation. According to Shaffer’s classification, if the ACA is greater than 20°, there is no potential for angle-closure; if the angle is narrower than 20°, there is a risk of angle-closure. Therefore, combined with the changes in ACA before and after ICL surgery, when the ACA before ICL implantation is less than 40° or less than 20° after surgery, or when the vault is high, we should be aware of the risk of ACA closure or glaucoma after surgery.

The inter- and intra-observer agreement of the ITC index for measuring the degree of angle-closure using SS-OCT has been reported to be good [17, 18]. The index showed moderate agreement and good diagnostic performance for angle-closure with gonioscopy as the reference standard, with a sensitivity of 71.9% and specificity of 84.3% [19]. We found that the ITC index was positively correlated with the vault after ICL implantation but not with the postoperative angle parameters or the percentage change in angle parameters. In this study, the highest vault was 1.201 mm and the highest ITC index was 22.2% 1 month after ICL implantation. In all patients no significant increase in IOP or glaucoma was found after surgery, which may be related to the change in the anatomical angle earlier than the change in IOP, or the degree of angle closure not decreasing below the anatomical threshold [20]. A greater ITC may be a biomarker for higher IOP and greater visual field loss [21]. In evaluating the ITC index in the primary angle-closure disease population, Barkha et al. [22] found that the ITC index in the primary angle-closure suspect, primary angle, and primary angle-closure glaucoma groups were 10.76%, 28.53%, and 81.43%, respectively. Therefore, in this study, we further employed ROC curves to identify the performance of the vault for identifying eyes with suspected angle-closure. We found that vault of > 0.659 mm was optimal for eyes with angle-closure suspect with a sensitivity of 85.2% and a specificity of 53.9%, which was lower than the upper limit of optimal vault recommended by Starr Surgical. Therefore, despite the postoperative vault ≤ 750 μm, there is still a risk of angle-closure when the vault is at a normal high value.

The safety of ICL implantation surgery has always been a focus of attention, and an appropriate vault is an important guarantee of the safety of ICL surgery. When the ICL is placed in the ciliary sulcus, its posterior “pushing” mechanism on the iris results in secondary angle closure and peripheral iris obstruction, impeding aqueous humor outflow and leading to elevated IOP. According to our findings, an excessive vault may result in a decrease in ACA width and an increase in the ITC ratio, which may cause the angle to close. Studies have shown that 6.08–56.0% of PACS patients will progress to PAC or PACG within 5 years [23–25]. In addition, the eyes of Asians and women are considered to have a genetic and anatomical tendency for angle closure [26, 27]. Wei et al. [28] found that 10 eyes (0.10%) underwent secondary surgery due to complications of excessive vault (range, 730–1190 μm) in 10,258 eyes that underwent ICL V4c implantation surgery for an average of 20 months. Hu et al. [29] also reported a case of high intraocular pressure and secondary glaucoma due to angle narrowing (Shaffer II-III grade) after ICL implantation, and the postoperative vault was only 456 μm. Moreover, ophthalmologists usually use the STAAR manufacturer’s online calculator to determine ICL size. This method is based on the preoperative WTW and ACD of the patient, and the indicated ICL size is generally too large, resulting in a high proportion of patients with an excessive postoperative vault [30]. Therefore, more attention should be paid to angle narrowing and suspicious angle-closure caused by an abnormal vault after ICL implantation.

This study had some limitations. First, the number of patients was relatively small and most patients were female. Although the SS-OCT examinations were performed indoors under the same light intensity, we did not consider the effects of darkrooms and eye accommodation movements on the vault and angle structures. Additionally, both eyes were included in the study and there was some symmetry in the anterior chamber anatomy of both eyes in the same patient [31]. It is important to continue monitoring changes in ICL implantation on anterior segment structures over an extended period in a large multicentre patient cohort. In addition, novel anterior segment parameters may have added value to this observation.

In conclusion, we observed a decrease in ACA parameters 1 month after ICL implantation, and their percentage change correlated with the vault. This is the first study to explore the risk of angle-closure after ICL implantation using the ITC index in a population using PACS and ROC curves. When the vault is greater than 0.659 mm, angle-closure should be suspected.

## Data Availability

The datasets used and analysed in the current study are available from the corresponding author upon reasonable request.
